# International comparison of trends in cancer mortality: Japan has fallen behind in screening-related cancers

**DOI:** 10.1093/jjco/hyab139

**Published:** 2021-08-31

**Authors:** Kota Katanoda, Yuri Ito, Tomotaka Sobue

**Affiliations:** Division of Surveillance and Policy Evaluation, Institute for Cancer Control, National Cancer Center, Tokyo, Japan; Department of Medical Statistics, Research & Development Center, Osaka Medical and Pharmaceutical University, Takatsuki Japan; Division of Environmental Medicine and Population Sciences, Graduate School of Medicine, Osaka University, Suita, Japan

**Keywords:** cancer control, mortality, neoplasms, population surveillance, vital statistics

## Abstract

While the age-standardized mortality rate in Japan is decreasing for all cancers as a whole, this is not the case for some major site-specific cancers. We descriptively compared trends in all-cancer and site-specific cancer mortality in Japan and selected countries. Data on age-standardized cancer mortality rates in six countries (Japan, the USA, the UK, Canada, Australia and the Republic of Korea) in 1980–2016 were obtained from the World Health Organization mortality database. While stomach and liver cancer mortality rates in Japan and Korea were initially much higher than those in non-Asian countries, they have rapidly decreased over the long term. By contrast, colorectal, pancreatic and cervical cancer mortality rates in Japan, which were initially lower than those in other countries, have increased such that they are now similar or higher than the rates in non-Asian countries. For male lung cancer, Japan’s initially lower mortality rate is now comparable to that in non-Asian countries as a result of slower decline. Meanwhile, the mortality rate of female breast cancer in Japan and Korea has increased and is nearing the rates observed in non-Asian countries, which by contrast have shown a steady decrease. Thus, while Japan has been successful in reducing the burden of stomach and liver cancers, it is falling behind in reducing the mortality rate of screening-related cancers such as colorectal, female breast and cervical cancers. Control measures for these cancers need to be strengthened.

## Teaser

Although Japan has been successful in reducing burden due to stomach and liver cancers, it faces challenges related to reducing screening-related cancers, namely, colorectal, female breast and cervical cancers.

## Introduction

Trends in cancer mortality are essential for monitoring cancer control (1). Extensive literature is available on global and national trends in cancer mortality, including in Japan, most of which report findings for site-specific cancers (2–6) or specific countries or regions (7–15). A recent study in Japan reported that some major cancers have not seen a favorable decrease in mortality (13). However, this phenomenon has not been examined in an international context. Here, we aimed to descriptively compare trends in all-cancer and site-specific cancer mortality in Japan and selected countries.

## Materials and methods

Data on age-standardized rates (ASRs) of all-cancer and site-specific cancer mortality in each country were obtained from the World Health Organization (WHO) mortality database (16). ASRs were adjusted to the world standard population (Segi) (17). We compared the status in Japan with that in the USA, the UK, Canada, Australia and the Republic of Korea (Korea). These countries were selected from the North America, Europe and Western Pacific regions. We analyzed all cancers and nine major site-specific cancers: stomach [International Classification of Diseases, 10th revision (ICD-10) code: C16], colon/rectum/anus (C18-C21), liver (C22), pancreas (C25), lung (C33-C34), female breast (C50), prostate (C61), cervix uteri (C53) and corpus uteri (C54). ASRs were calculated for two age groups: all ages and ages <75 years old. We examined the latter because national cancer control plans in several countries, including Japan, have chosen the ASR in those <75 years of age as a numerical goal. Further, this parameter is useful for investigating the effect of cancer control interventions such as screening (18–20). Crude mortality rates were also calculated for the two age groups to examine results, including the effect of aging. The observation period was basically set to 1980–2016. Though data were available from 1950s for all countries except Korea, we limited our analysis to 1980 and onward because causes of deaths other than cancer, such as neonatal and communicable disease mortality, were considered to have affected the earlier period. The observation period in each country was dependent on the available data for each cancer in the database: 1980–2015 for Canada, 1982–2016 in Australia for liver cancer and 1985–2016 for Korea (1992–2016 for liver cancer and 1995–2016 for cervical cancer). In the case of liver cancer in Japan, we used only data from 1995 and later because the definition of liver cancer changed in 1995 following an update of the ICD.

## Results

**Figure 1:**
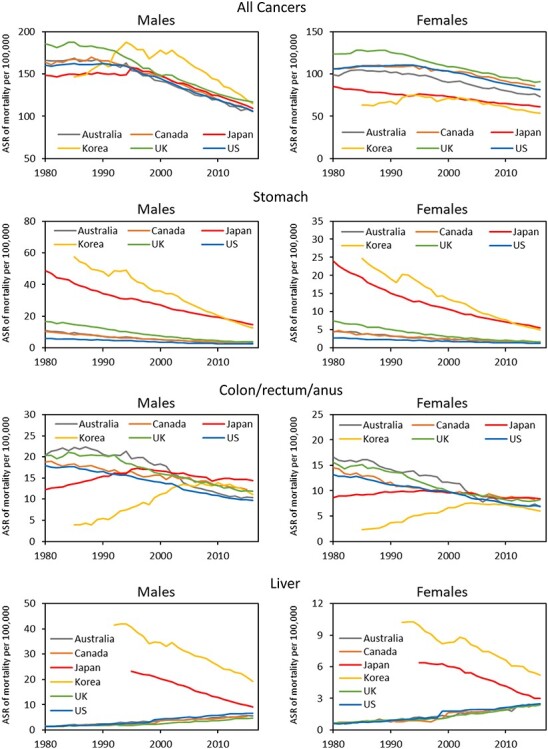

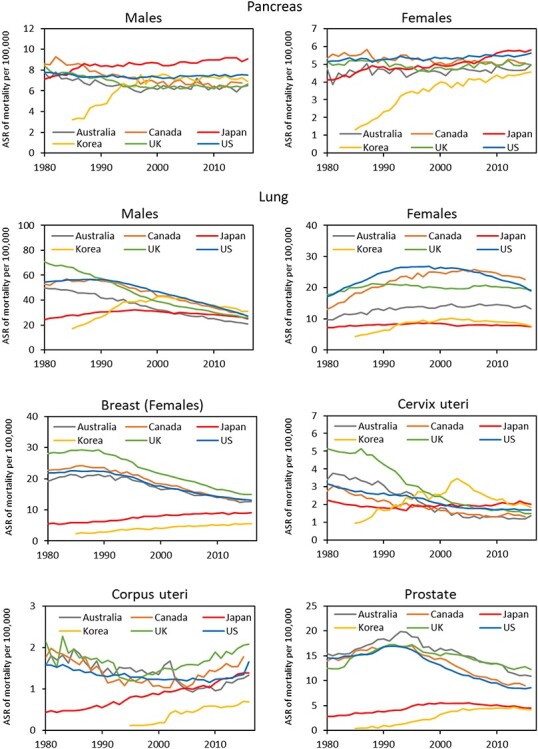
The trends in the ASR of all-cancers and site-specific cancer mortality by sex and country for all ages.

Figure 1 shows trends in the ASR of all-cancer and site-specific cancer mortality by sex and country for all ages. In Japan, the ASR of all-cancer mortality combined, for males, showed a similar trend to that in the UK, USA, Canada and Australia: the mortality rate decreased from 1990s at a comparable rate and to comparable absolute values. Korea showed a steeper increase in mortality rate in the 1990s and a steeper decrease after 2000 compared with the other countries. For females, the mortality rate in Japan has steadily decreased throughout the observation period, with absolute values remaining lower than those in the USA, UK and Canada. The mortality rate in these countries as well as Australia started to decrease from the 1990s at a rate close to that observed in Japan. Korea also showed decreasing mortality from the 1990s, albeit at a slightly faster rate than Japan.

For stomach cancer, the mortality rate in both males and females was more than 2-fold higher in Japan and Korea than in non-Asian countries. In Japan and Korea, however, the rate decreased steeply, with Korea showing a steeper decline than Japan, culminating in lower absolute rates in Korea in recent years. Mortality rates in non-Asian countries also steadily decreased through the observation period.

In the 1980s, the mortality rate of colorectal cancer (including anal cancer) in both males and females in Japan was approximately two-third of that in the USA, UK, Canada and Australia. However, the rate increased steeply until the 1990s to reach levels comparable to those in non-Asian countries. While the mortality rate in Japan decreased thereafter, that in the four non-Asian countries decreased even more steeply from the 1980s. As a result, the mortality rate in Japan has been the highest among all countries examined in recent years. While Korea also showed a steep increase in mortality rate in the 1990s, it experienced a steeper decline than Japan thereafter, resulting in rates similar to or lower than those in non-Asian countries.

The mortality rate of liver cancer in both males and females in Japan was initially much higher than that in the USA, UK, Canada and Australia but steadily decreased through the observation period. By contrast, the mortality rate in the four non-Asian countries increased throughout the observation period, approaching the rate observed in Japan. The mortality rate in Korea has decreased in an almost parallel manner to that in Japan and remains at a slightly elevated level.

For pancreatic cancer, the mortality rate in both males and females in Japan was at lower level than that in non-Asian countries during the first phase of each observation period. After that, the rate rapidly increased during the 1980s and continued to slowly increase thereafter, reaching a higher level than that in non-Asian countries.

For lung cancer in males, the mortality rate in Japan increased, peaking in around the mid-1990s and slowly decreased thereafter. Similar patterns were observed in USA and Canada, while UK and Australia showed a steady decreasing trend throughout the observation period. The decline in mortality rate was steeper in the non-Asian countries than in Japan. Consequently, absolute rates in the countries examined, which were initially lower in Japan than in non-Asian countries, have reached similar levels. Notably, the mortality rate in Japan was almost the same as that in the UK in the most recent year examined. In Korea, the mortality rate peaked at around the year 2000, and the most recent value was the highest among all countries examined. In females, the mortality rate in Japan increased until the 1990s before slowly decreasing thereafter. Similarly, the mortality rate in the other countries increased in the early phase of the observation period before decreasing or stabilizing thereafter. The mortality rate in Japan and Korea has remained lower than that in the other countries.

For female breast cancer, while the mortality rate increased continuously in Japan and Korea throughout the observation period, it remained lower than those in the four non-Asian countries. By contrast, the mortality rate in the non-Asian countries has been rapidly decreasing since the 1990s.

For cancer of the cervix uteri, the mortality rate steadily decreased during the first phase of the observation period in all countries except Korea. Subsequently, however, the mortality rate in Japan started to increase, while those in the other countries continued to decrease. The mortality rate in Korea rapidly increased until the 2000s before reversing to a rapid decrease. As a result, Japan has had the highest mortality rate in recent years, even higher than that in Korea.

The mortality rate of cancer of the corpus uteri in Japan and Korea increased continuously during the observation period. In recent years, the mortality rate in Japan has reached a similar level to that observed in the USA and Australia. The mortality rate in the four non-Asian countries decreased until the 1990s before increasing thereafter.

For prostate cancer, the mortality rate in Japan and Korea continuously increased until around the year 2000 and decreased slowly or remained stable thereafter. By contrast, the mortality rate in the four non-Asian countries gradually increased until the 1990s, showing a spike-like increase around 1990, before decreasing thereafter. The slopes of the increases and decreases in mortality rate were steeper in the four non-Asian countries than in the two Asian countries.

Supplementary Figure S1 shows the corresponding results for individuals aged <75 years old. The patterns were closely similar to those observed for all ages. Supplementary Figures S2 and S3 show the corresponding results for crude mortality rate among individuals of all ages and aged <75, respectively. Individuals in both age groups in Japan showed marked increases in crude mortality rates, particularly for colorectal, pancreatic, male lung and cervical cancers. In fact, Japan has had the highest crude mortality rate for these cancers among the countries examined in the last several decades. For female breast cancer, there was a sharp contrast between the decreasing trend in non-Asian countries and the increasing trend in Japan and Korea.

## Discussion

Here, we performed a descriptive comparison of the trends in all-cancer and site-specific cancer mortality in Japan and selected countries. Although the all-cancer mortality rate in Japan has been decreasing in a similar manner to those in other countries, trends in site-specific cancers have differed. Stomach and liver cancers have shown favorable trends: the initially high mortality rate of these site-specific cancers in Japan (and in Korea), compared with non-Asian countries, has rapidly decreased over the long term. By contrast, however, the initially lower mortality rate of colorectal and pancreatic cancers and cancer of the cervix and corpus uteri in Japan, compared with non-Asian countries, increased rapidly during the observation period and reached a similar or higher level than that observed in non-Asian countries. For male lung cancer, the decrease in mortality rate in Japan has been less steep than that in other countries, leading to a comparable absolute rate of mortality between Japan and the other countries examined. While the mortality rate of female breast cancer remains lower in Japan, it is approaching that observed in non-Asian countries, which, unlike Japan, have shown a clear decreasing trend. Notably, these same unfavorable trends were also evident in the crude mortality rate, indicating a direct increase in cancer burden in the Japanese population.

A series of papers by Katanoda et al. has analyzed the trends in cancer mortality in Japan (12–14). The long-term decrease in the mortality rate of stomach cancer is thought to be due to the decline in the prevalence of *Helicobacter pylori* infection combined with improvements in sanitation, diet (reduced salt intake) and food preservation techniques (21,22). Early detection and improvements in prognosis after 2000 are also contributing factors (23,24). While coverage of *H. pylori* eradication by the national health insurance scheme from 2013 may also have contributed to the decrease in mortality (25), the effect is currently unclear (13). The mortality rate of stomach cancer in Korea has decreased more rapidly than in Japan. This may be due to the fact that Korea has established intensive secondary prevention measures for stomach cancer, mainly using endoscopy. Indeed, the participation rate has increased annually by 4.4% from 2004, reaching 72.8% in 2018 (26).

The rapid decrease in liver cancer in Japan has been attributable to the decline in the prevalence of hepatitis virus (HV) infection (mainly HCV) (27). The availability of improved therapeutics from the early 2000s, such as pegylated interferon in 2004 and direct acting antivirals in 2011, is also thought to have contributed to the decrease in incidence and mortality (28–30). Together with stomach cancer (31), liver cancer is an example of a successful control measure implemented in Japan for infection-related cancers (32–34).

By contrast, however, Japan has been falling behind other countries in control measures for colorectal, female breast and cervical cancers. There is sufficient worldwide evidence that population-based cancer screening is effective in reducing the mortality rate of these cancers (1,35–38). Indeed, the reduction in colorectal, and female breast cancer mortality in the USA has been shown to be largely attributable to the dissemination of cancer screening (39,40). The participation rate in cancer screening in Japan has been lower than that in other countries (41). Dissemination of cancer screening with proper quality control is thus urgently needed to reduce colorectal, female breast and cervical cancers in Japan.

In addition to cancer screening, cervical cancer can also be prevented through human papillomavirus (HPV) vaccination (42). Although it will take several decades to observe a reduction in the cervical cancer mortality rate as an effect of vaccination coverage, several countries that introduced the vaccine earlier have already observed a reduction in invasive cervical cancer incidence (43–45). In Japan, accumulating evidence is demonstrating that the effectiveness of the HPV vaccine is consistent with that reported in the international literature for reducing HPV infection and cervical precancerous lesions (46–49). However, the Japanese government’s halting of its proactive recommendation of the national HPV vaccination program, which was caused by fear of potential adverse effects in 2013, has yet to be lifted. As a result, generations born after the year 2000 have missed the opportunity to receive the vaccine (50). Some local municipalities have started to send information or recommendation for the vaccination program to eligible women. Further actions are urgently needed at the national level (51).

The lung cancer mortality rate in Japanese males has been steadily decreasing since the mid-1990s, a result that has been attributed to the decline in smoking prevalence and improvement in prognosis for patients with chemotherapy (13,52,53). However, according to the latest data, the male smoking prevalence in Japan is 27.1% (2019) (54), remaining higher than that in the non-Asian countries analyzed in the present study (around 20% or lower) (55). Comprehensive tobacco control measures in line with the WHO Framework Convention for Tobacco Control and MPOWER policy package are needed (56–58).

Pancreatic cancer is known to have one of the poorest prognoses of all cancers (59). The mortality rate of this cancer has continued to increase in Japan and has surpassed the rate observed in the other countries analyzed in the present study. Although the reason for this increase is unknown, evidence suggests that an increase in risk factors, such as type 2 diabetes (60), and improvements in diagnostic measures, such as computed tomography imaging and biopsy for histologic confirmation (61), have made a contribution to the long-term increase during the period including years earlier than those in our analysis (13). Continued efforts are needed to promote preventive measures for known risk factors (e.g. tobacco smoking and type 2 diabetes) (62) and to develop novel technologies for early detection and effective treatment.

In summary, our international comparison of trends in cancer mortality rate show that while Japan has been successful in reducing the burden of infection-related stomach and liver cancers, it continues to face major challenges in reducing screening-related cancers such as colorectal, female breast and cervical cancers. Further efforts are also needed to manage lung and pancreatic cancers.

## Supplementary Material

SuppFig1_ASR_U75_0729_hyab139

SuppFig2_curde_rate_all_0729_hyab139

SuppFig3_crude_rate_U75_0729_hyab139
